# The Challenge of Data Annotation in Deep Learning—A Case Study on Whole Plant Corn Silage

**DOI:** 10.3390/s22041596

**Published:** 2022-02-18

**Authors:** Christoffer Bøgelund Rasmussen, Kristian Kirk, Thomas B. Moeslund

**Affiliations:** 1 Visual Analysis and Perception Lab, Aalborg University, Rendsburggade 14, 9000 Aalborg, Denmark; tbm@create.aau.dk; 2 CLAAS E-Systems, Møllevej 11, 2990 Nivå, Denmark; kristian.kirk@claas.com

**Keywords:** deep learning, dataset, annotation, semi-supervised learning, whole plant corn silage

## Abstract

Recent advances in computer vision are primarily driven by the usage of deep learning, which is known to require large amounts of data, and creating datasets for this purpose is not a trivial task. Larger benchmark datasets often have detailed processes with multiple stages and users with different roles during annotation. However, this can be difficult to implement in smaller projects where resources can be limited. Therefore, in this work we present our processes for creating an image dataset for kernel fragmentation and stover overlengths in Whole Plant Corn Silage. This includes the guidelines for annotating object instances in respective classes and statistics of gathered annotations. Given the challenging image conditions, where objects are present in large amounts of occlusion and clutter, the datasets appear appropriate for training models. However, we experience annotator inconsistency, which can hamper evaluation. Based on this we argue the importance of having an evaluation form independent of the manual annotation where we evaluate our models with physically based sieving metrics. Additionally, instead of the traditional time-consuming manual annotation approach, we evaluate Semi-Supervised Learning as an alternative, showing competitive results while requiring fewer annotations. Specifically, given a relatively large supervised set of around 1400 images we can improve the Average Precision by a number of percentage points. Additionally, we show a significantly large improvement when using an extremely small set of just over 100 images, with over 3× in Average Precision and up to 20 percentage points when estimating the quality.

## 1. Introduction

Monitoring the harvesting of Whole Plant Corn Silage (WPCS) with a forage harvester can enable a farmer to react to varying conditions by altering key settings in their machine in order to maximise quality. Current approaches used by farmers are mostly based on manual sieving of samples, which gives information on the particle size distribution. However, recent works [1,2] have shown the promise of using deep learning in the form of Convolutional Neural Networks (CNNs) for automatic object recognition in samples taken directly from the machine. These methods have minimal manual steps, allowing farmers to efficiently react in the field. However, the usage of CNNs introduces challenges in creating image datasets, as it is widely known that models require large amounts of annotated data to train [3]. Large datasets such as ImageNet [4] and COCO [5] have been one of the key reasons for the progression in computer vision over the past decade. Quality and consistency of the annotations is key, and often this is acquired through well defined multi-stage processes including team members who take on different roles. Naturally, this can be a time-consuming and expensive process. Alternative or additional methods can also be used to speed up the manual process, including approaches such as transfer learning, weak supervision, or Semi-Supervised Learning (SSL) [6]. These data and annotation challenges exists in other agricultural use-cases [7,8] but also in other domains including nano particles [9], rock fractures [10], or medical images [11].

**Figure 1:**
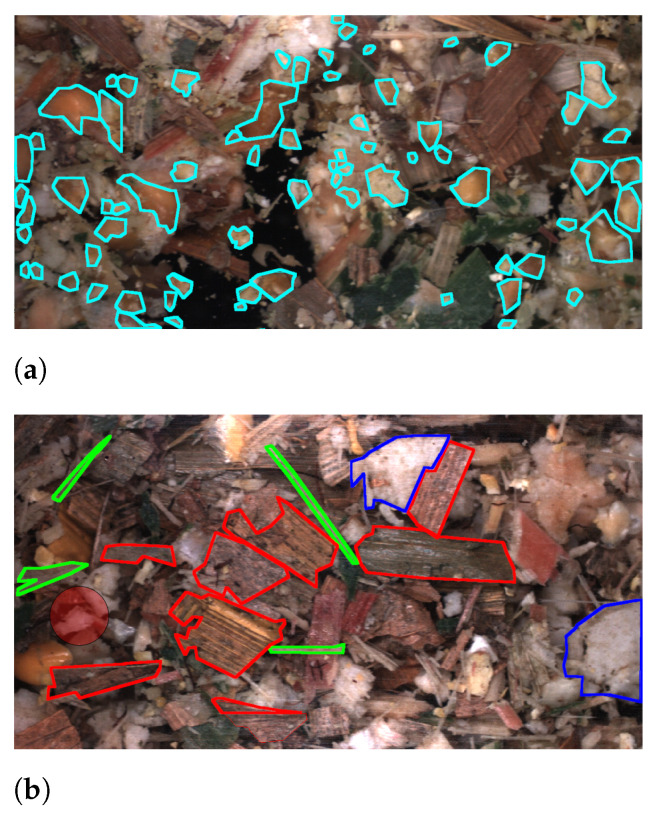
Example annotations of kernel fragments (**a**) and for stover overlengths in (**b**).

The quality of the harvested crop is highly dependent on farmers using correct machine settings for their harvester to react to their field conditions [12]. Two of the key settings are the Processor Gap (PG) and Theoretical Length of Cut (TLOC), which primarily affect the fragmentation of kernels and chopping of stover particles, respectively. The PG is the gap between rotating processor rolls that compresses and cracks kernels into fragments. The TLOC is controlled by the speed of a rotating knife drum, where a higher speed chops the plant into smaller particles. In Figure 1 examples from our two forms of datasets are shown. Figure 1a shows an example of kernel fragment annotations. In this case our aim is to create an annotated dataset containing instances of kernel fragments such that we can train a network to perform object recognition and thereby estimate the quality across images. For quality, we estimate the industry standard metric Corn Silage Processing Score (CSPS) [13] which gives a measurement of the percentage of kernel fragments passing through a 4.75 mm sieve. A higher CSPS indicates higher quality, since the kernels are easier to digest when the WPCS is used as fodder for dairy cows. Figure 1b shows annotations of stover overlength annotations. For kernel fragments, the aim was to annotate and predict all instances, however, this task was deemed to be too demanding for stover particles as all remaining instances would have to be marked. Therefore, we only annotated particles marked as overlengths, which are classified based on how the WPCS was harvested. Farmers can have different strategies for the chopping of stover particles given their requirements. For example, longer particles can promote cud chewing but shorter particles can be easier to pack in a silo [14]. Therefore, we annotate such that we can measure a dynamic overlength given the farmer’s chosen TLOC. This overlength definition is 1.5 × TLOC. The WPCS in Figure 1b is harvested with a TLOC of 4 mm and therefore particles greater than 6 mm are annotated. Additionally, for stover annotations we annotated four classes covering different parts of the plant. Figure 1 shows that for both datasets the instances are challenging for both a network to predict but also for annotators to annotate due to the high amounts of clutter between particles.

While highly-defined processes can lead to a high quality dataset, it can be an expense that is not available in all projects, especially in the early phases. This has been the case for our datasets for WPCS, which have been used in a number of works [1,2,15]. However, they have shown to produce promising results. Therefore, in this work we investigate the challenges of data annotation for deep learning. This includes presenting our processes for creating an WPCS image dataset with annotated object instances through manual annotation. We show our guidelines for annotating datasets leading to supervised learning with CNNs. The resulting datasets and models show that the methodology is viable, however, as the datasets scale to larger sizes through multiple annotators, the consistency falters. Annotator disagreeance is a common challenge which can be addressed with well-defined processes [3], however, this is costly to create and manage. Alternatively, the field of SSL aims to take advantage of more efficiently gathered higher-level or noisy input to train models [6] which we evaluate for our purpose. While extensive literature exists for the process of creating datasets for larger benchmarks, it is limited in more specific agriculture-based works. Therefore, our aim is to show and evaluate our approach, including the challenges in building datasets for data-driven machine learning.

Presenting the challenges of the annotation process in specific tasks is important in order to evaluate the usage of data-driven models. Before our previous works [1,2,15], the works presented on monitoring WPCS harvest with computer vision have been conducted with classic computer vision, which have not required annotations for algorithm development. In [16], CSPS was estimated by finding the contours of kernel fragments, which were manually separated from the sample and spread out on a black background. For stover quality estimation, a number of works have also used feature engineering to determine the mean particle length of separated and spread out particles [17,18]. Our previous publications were the first to tackle WPCS monitoring with both deep learning and in non-separated samples covering the annotation process, which allows other researchers to understand the problem further and manoeuvre around potential pitfalls.

Our contributions in this work are therefore threefold:

- Present our annotation process for WPCS with respect to kernel fragmentation and stover overlengths;
- Show an analysis of the quality and consistency of the resulting annotations;
- Evaluate SSL for WPCS, showing a considerably more efficient alternative to manual annotations for supervised learning.

## 2. Related Work

To the best of our knowledge there do not exist any image datasets for WPCS. Therefore, we investigate dataset creation in regards to benchmark datasets for both agriculture and in general object recognition. Starting with the latter, there exists a large number of public datasets in the computer vision domain. For example, paperswithcode (Available online: https://paperswithcode.com/datasets accessed on 1 December 2021) lists 160, 195, and 37 for object detection, semantic segmentation, and instance segmentation, respectively. Larger benchmark datasets have the ability to form golden standards in the computer vision community and can be used to evaluate algorithms and push overall research.

Common among them is the aim to have a dataset with high quality and consistent annotations, often over hundreds of thousands of images and hundreds of potential classes. The process for creating such datasets is expensive and therefore requires an efficient and clear pipeline. Typically a team of workers, either internal or outsourced, are instructed to annotate following a multiple stage pipeline aimed to maximise consistency and coverage. For example, in the ImageNet [4] object detection challenge, a multi-stage solution first determined which object classes were present in a given image using a query-based algorithm to quickly traverse the 200 potential classes. Given these image-level class definitions, an annotator is given a batch of images and instructed to draw a single bounding-box before moving to the next image. An image continues in this process until all bounding-boxes are annotated. Bounding-box quality and coverage are iteratively checked by another worker and once both pass the image is accepted into the dataset. Another multi-stage example is for the instance segmentation annotations in COCO [5] where images are annotated in three steps. First, an annotator determines if an object instance is present from a number of pre-defined super-categories—if yes, a symbol for a each specific sub-category is dragged and placed on a single instance. Next, each instance of every sub-category is marked until all object instances are covered. Finally, instance segmentation masks are annotated for each of the marked instances. During the final stage, an annotator is asked to only annotate a single mask. Additionally, they are informed to verify previous segmentation annotations from other workers. In LVIS [19] the creators adopt a similar iterative pipeline to COCO of first spotting single object classes per image, followed by exhaustively marking each instance of a given category. In the next stage, instance markings are upgraded to segmentation masks before moving on to verification. In a final stage, negative labels are added to the image. A three stage approach is used in Objects365 [3], where first non-suitable iconic images are filtered. Iconic images typically only have a single clear object in the middle of the image and are deemed to be too simple. Next image-level tags are added based on super-categories, followed by the final step of annotating all bounding-boxes into sub-categories. There are also examples of creating datasets through less-defined processes and rather attempt to conduct the annotations closer to the expert knowledge, however, this is less common as the size of datasets are becoming increasingly larger. For example, in PASCAL VOC [20], the initial annotations were done by researchers at a single annotation event. While in ADE20K [21] the dataset is ambitiously annotated by a single person, aiming to maximise consistency.

Common to most benchmark datasets is the usage of various roles that often require training. The role of an annotator is naturally used in all benchmarks and a training task is given to evaluate their ability. For example, in ImageNet [4], annotators must pass a drawing and quality verification test. In both tests the aim is to learn three core rules, for example, for drawing boxes only the visible parts should be annotated as tightly as possible. Multiple roles can add further verification, such as in Objects365 [3]. Here, a course must be taken to learn how to become an annotator or inspector. Annotators are trained to draw bounding-boxes and inspectors are trained to verify all annotated images. Furthermore, an examiner role is also included to review output from the inspector.

Finally, the usage of a golden standard set, where annotations are verified by experts to be near 100% accurate are used throughout almost all of the benchmarks. In LVIS [19], gold sets are added in multiple places in the pipeline and further work is prioritised to reliable workers. In ImageNet [4] they are used as overall quality control and also during training of annotators and inspectors.

While procedures such as multi-stage pipelines, training, and roles can be important, there also exists alternative approaches to either aid annotators or speed-up the tasks. This can be especially useful if dataset creators do not have a large amount of resources to implement the points covered so far. Researchers have investigated how to make the process of drawing annotations on an image more efficient. For example, Extreme Clicking was introduced in [22] and was used to annotate the Open Images dataset [23]. Extreme Clicking allows for fast drawing by having the user click the four most extreme points of an object. It was found to decrease the drawing time from 25.5 s to 7.4 s in comparison to traditional box dragging. Annotation tools can also be enhanced by allowing the tool to produce annotations that a user can adjust [24,25,26]. Active learning is an approach that in itself does not improve the actual annotation but can aid by determining which set of images should be annotated next in order to more efficiently improve the model. This can be achieved by determining the uncertainty on a number of unlabelled image and prioritising them for annotation [27,28]. Another alternative is weak supervision, which takes lower quality labels and is able to transfer this knowledge into the training. Such approaches use features from models pre-trained on larger datasets to train a classifier, or models can be finetuned towards a more specific task [6].

Data augmentation is a method that is widely used across training of deep networks, where the general approach is to alter already annotated images to vary the input data. This can include transforming the image such as flipping or cropping, colour transformations, blurring the image, or even removing parts of the image. Newer approaches have increased the complexity of augmentation by aiming to learn new representations. This can include using Generative Adversarial Networks to produce synthetic data [29]. Another approach is to use neural network-based augmentation networks to learn the best strategy for applying augmentation. Popular approaches include Auto Augment, which searches for the best augmentation for a specific image [30].

A popular approach in SSL is to increase the amount of labelled data by using pseudo labels from a fully-supervised model, where a teacher network trains a student network. This approach has been popular in classification tasks, but less so in object detection and segmentation, as the latter tasks are often more challenging due to the often large class imbalance between background and foreground objects [31]. However, recent works exist that aim to take these and additional challenges into account [31,32].

Within agriculture there exists a number of datasets for different applications. These are extensively covered in [33] and we use this work as inspiration to analyse the dataset creation in similar applications to our work. For most agriculture dataset papers there is minimal description of the process of conducting annotation. Most simply state that object instances were annotated in either a bounding-box or mask format. In some cases there is a description of an open-source annotation tool but without stating details, e.g. the MangoNet dataset [34]. However, a few provide details on the specific tool, including DeepSeedling [35], where a dataset of bounding-boxes for cotton seedlings is collecting using MS VoTT. Additionally, in the MineApple dataset [36], the VIA annotation tool is used to annotate apples with bounding boxes. Finally, in DeepFruits [37], a custom MATLAB annotation tool is produced, which has been publicly released by the authors.

As mentioned, the process for collecting and conducting annotation is rarely covered apart from a couple of datasets. An exception is the MineApple dataset [36], here an annotation worker is first instructed how to annotate before they can perform the task, and, after annotating, an initial 10 images are given in-person feedback. Furthermore, verification of all annotations is done to correct annotations from the workers. The process is also briefly described for annotating corn tassels in [38], where annotators are given a training page before starting and gold standard sets are used to evaluate the resulting annotations.

Lastly, a number of the datasets adopt tools that counteract manual annotation. In the Orchid fruit dataset [39], a custom tool is able to train and test in parallel during annotation, allowing to easily determine changes in accuracy as additional examples are added to the dataset. In the Fruit Flowers dataset [40], the annotation tool FreeLabel [41] is aided by having the worker draw freehand on a tablet for regions that contained flowers and the tool generated masks using region growing refinement. Finally, synthetic annotation has been used for the GrassClover dataset [42], by pasting plant crops onto background images of soil while randomly sampling rotation and scale in addition to adding shadows to the crops.

## 3. Dataset Annotation

In this section we present an overview of our process for creating annotated datasets for WPCS. Two different forms of dataset are created: one for kernel fragmentation and another for stover overlengths. For each dataset, we cover our annotation guidelines for annotators, present statistics over datasets, and present an evaluation of the quality and consistency of annotations.

### 3.1. Kernel Fragmentation

As already mentioned, the datasets for kernel fragmentation have been previously used in a number of works [1,2,15]. The works showed for a number of deep learning models the potential of measuring kernel fragmentation in non-separated samples. In [2] the trained models were additionally evaluated against physically sieved samples for CSPS, showing a strong correlation. In these works, fully supervised deep learning models in the form of bounding-box detectors or instance segmentation networks were trained to localise all kernel fragments in an image of WPCS. For each predicted instance the size of the prediction was compared against the threshold of 4.75 mm, allowing for an estimate of CSPS. However, the best performing models between annotation-based metrics and CSPS correlation were not always consistent. Therefore, in this section we present and evaluate our process for annotating kernel fragmentation in our images.

#### 3.1.1. Annotation Guideline

**Figure 2:**
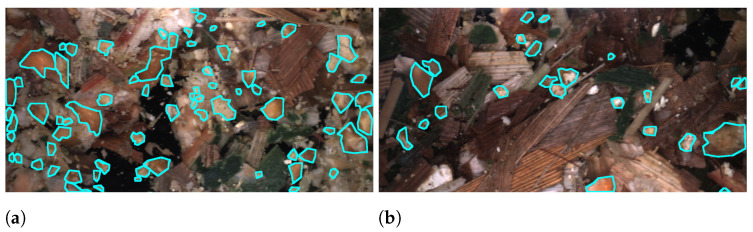
The difference in kernel fragmentation potentially present in images between different PGs. Both samples are harvested with TLOC of 11.5 mm but (**a**) had a PG of 1 mm and (**b**) 4 mm.

To solve the task of estimating kernel fragment quality, the aim was to annotate all fragments, allowing for an estimation of an industry standard such as CSPS. This would ideally allow a system to learn and estimate from images the differences in fragmentation given the condition present to a farmer’s field. Figure 2 shows fragment annotations in two cases with a clear difference in fragmentation. Both images are captured in the same field and have an identical TLOC but with different PGs. A PG of 1 mm in Figure 2a produces a larger number of smaller fragments and fragments in total compared to Figure 2b harvested with PG 4 mm. It is worth stating that there is not necessarily such a significant difference in fragmentation, however, the general expectation is a larger number of fragments with a smaller size as the PG decreases.

**Figure 3:**
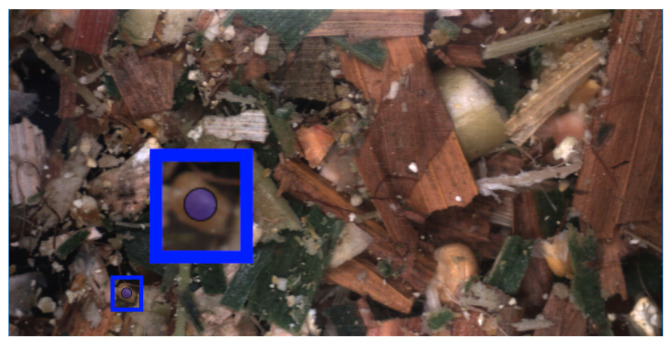
A blue indicator is shown, indicating the minimum size of particles to be annotated.

In addition to informing annotators to annotate all fragments, a number of specific cases were also addressed that occurred due to working with non-separated samples. Firstly, despite working with a resolution of 20 pixels to 1 mm, very small fragments in the images were both difficult to annotate and to determine whether they were truly kernel fragments. Therefore, an indicator was added to the annotation tool with a radius of 1 mm, showing the minimum size fragments should be before they are annotated. The indicator is shown in Figure 3 together with a zoomed in view. The indicator followed the user’s mouse cursor and if a fragment’s axis extended beyond the diameter the user should start the annotation process for the instance.

**Figure 4:**
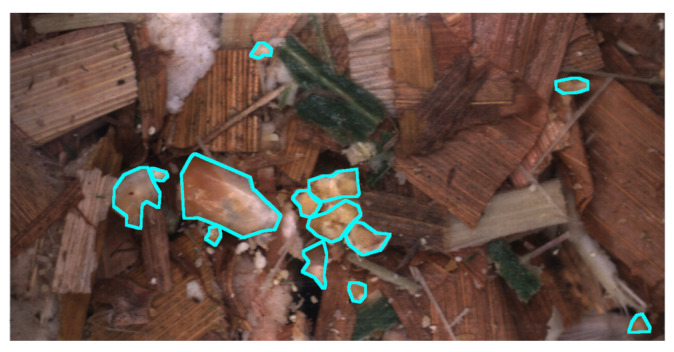
An example of how to annotate fragments that are grouped closely together.

Another specific case is when fragments are grouped closely. It could be ambiguous in these instances whether these were a single fragment or where the boundary between them should be. Therefore, a number of examples, such as Figure 4, was provided to annotators with the aim of providing guidance.

**Figure 5:**
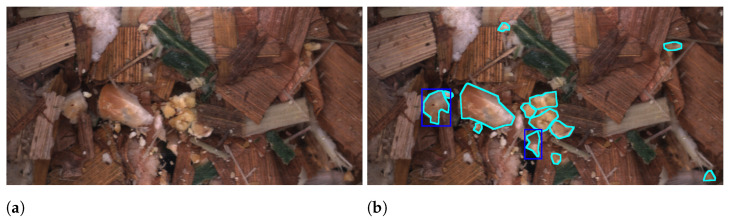
An example of how to annotate instances that are covered by other particles. Original image for reference in (**a**) and example annotation in (**b**).

Finally, as we are working with non-separated samples, kernel fragments can be partially covered by other fragments or stover. Naturally, this is not ideal as the image is not able to provide a true description of the fragmentation level for these cases. A solution could be for an annotator to estimate the true boundary, however, we determined this to be difficult and potentially lead to errors when training the data-driven models. Therefore, annotators were instructed to only annotate the visible boundary. This is visualised in Figure 5 with the original image in Figure 5a and two cases of annotations of covered fragments in Figure 5b.

#### 3.1.2. Statistics and Evaluation

The annotation process was conducted over a number of iterations as images were gathered over harvest seasons. Therefore, we have split the data into a number of datasets that are named based on the harvest year. These could be used either individually or combined for a larger dataset during model development. An overview of the annotation statistics for each harvest season can be seen in Table 1, showing the machine setting the silage was harvested with (PG and TLOC), the total number of images annotated, the total instances annotated, and the average instances per image. The statistics are summarised for each PG, as this machine setting has the largest effect on fragmentation within a dataset. Additionally, if there are multiple harvest sequences of the same PG, these statistics are summarised in a single row where the number in the parenthesis shows the total number of sequences. Firstly, we can see that 2017 dataset has a significantly larger number of total images and instances compared to the three other datasets. While the annotation process was completed over a number of years, a comprehensive effort was made after this harvest to build a large dataset, resulting in a skew towards this harvest. Secondly, the average number of annotated instances per image varies across the datasets, for example, between 2 to 8 instances in 2016 and 2017. Furthermore, a significant increase is seen in 2015 with 8 to 15 instances and in 2018 with 10 to 28 instances.

**Figure 6:**
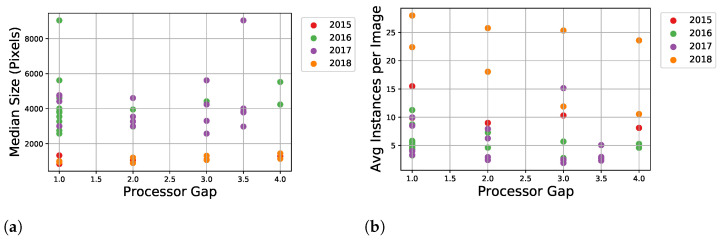
Median size of annotations for sequences across PGs (**a**). Average number of annotated instance for sequences across PGs (**b**).

**Figure 7:**
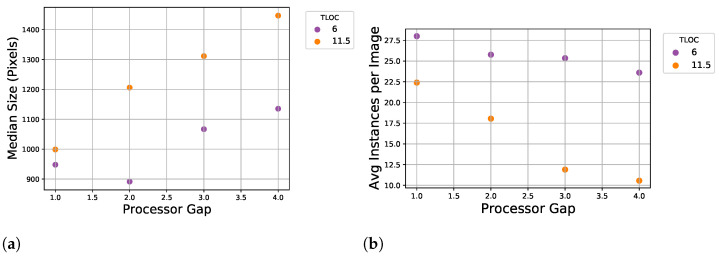
Statistics for annotations from 2018. Median size of annotations for sequences across PGs (**a**). Average number of annotated instance for sequences across PGs (**b**).

The differences in annotations are highlighted in Figure 6 with the average size of annotated instances (a) and average number of instances per image (b) for each sequence. The expectation, at least within a harvest year, is that in general a smaller PG should produce smaller and more fragments compared to larger PG. For the datasets from 2015, 2016, and 2017 this trend is not overly clear in Figure 6. However, the annotations from 2018 were done as a direct attempt to address this through a sanity check with a high requirement on annotation quality from a single annotator. This resulted in both a considerable increase in the average number of instances per image, as seen in Table 1 and a clearer trend over PGs in corresponding Figure 7a,b. Additionally, in these figures it can be seen the effect of the TLOC, where a shorter length affects fragments with smaller size and an increase in instances.

### 3.2. Stover Overlengths

In this section we cover the annotation process and statistics for determining stover quality. As covered in [2], we diverge from the kernel fragmentation strategy presented in the previous section and rather only aim to localise stover deemed as overlengths. An overlength per our definition is when a particle is 1.5 × TLOC or larger [2].

#### 3.2.1. Annotation Guideline

**Figure 8:**
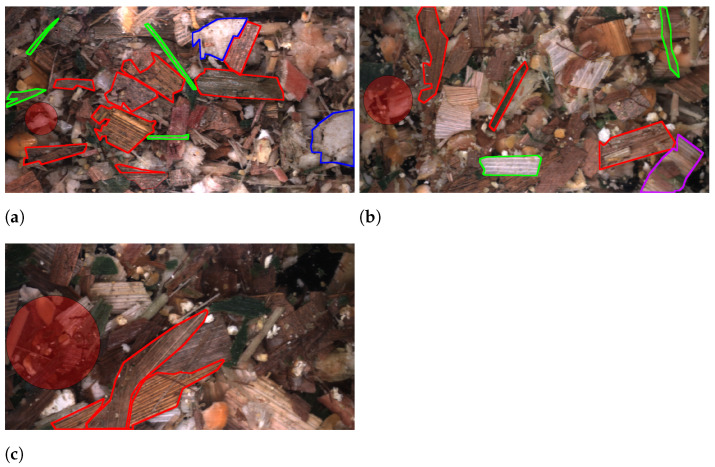
Differences in image content and annotations for three TLOC. In (**a**) samples are harvested with 4 mm, (**b**) with 6 mm, and (**c**) with 11.5 mm. For each image the overlength definition of 1.5 × TLOC is shown by diameter of the red circle.

The differing overlength definition was visualised to the annotators through a red circle indicator, as seen in Figure 8. The indicator could be used to see if an instance should be annotated based on whether it exceeded beyond the radius along any axis. The size of the red indicator is 1.5 × TLOC for a given image.

**Figure 9:**
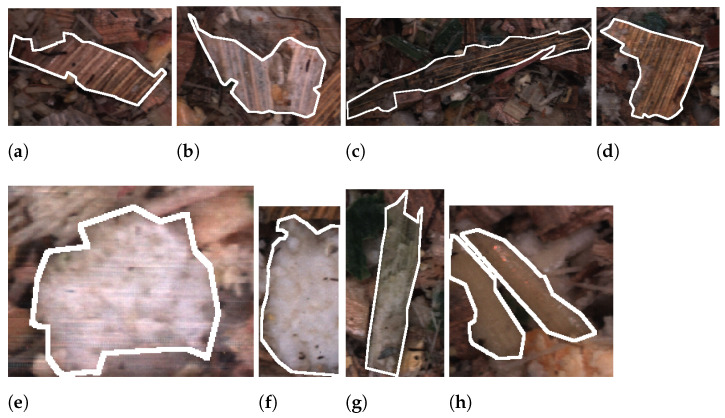
Overlength class examples, accepted leaves (**a**,**b**), non-accepted leaves (**c**,**d**), inner stalk (**e**,**f**), and outer stalk (**g**,**h**). Annotations examples are all from images captured of WPCS harvested at a TLOC of 4 mm.

In addition to informing how to annotate an overlength particle, the annotators were given similar instructions as those to kernel fragments. These include only annotating the visible portion of instances and annotating individual instances when multiple are tightly grouped. Finally, the annotators were given a number of example annotations aiming to cover both the inter- and intra-class variance. Figure 9 shows two examples of each class from image sequences captured at TLOC 4 mm. In Figure 9a,b the accepted leaves class is shown, which occurs when an instance is an overlength but only based on the axis length that is perpendicular to the leaf structure. In Figure 9c,d, the counterpart to the previous class non-accepted leaves is presented. In this case, the axis which follows the leaf structure exceeds the overlength definition. Figure 9e,f shows examples of inner stalks, which often has a sponge-like texture. Lastly, Figure 9g,h covers two examples of outer stalks where there can be some variance in the colour.

#### 3.2.2. Statistics and Evaluation

The final annotations used in [2] were done by a single annotator and an overview of the annotation statistics can be seen in Table 2. The table shows that in general there are more instances with a smaller TLOC, in addition to instances having a smaller size. Additionally, with the larger TLOC of 11.5 mm the annotations are limited for some classes, such as inner stalk.

Before defining the dataset shown in Table 2 and used in [2], an initial annotation iteration was done by three annotators on images harvested with a TLOC of 4 mm. As seen for kernels, we observe an inconsistency between the annotators on metrics such as the number of instances and average size, which we show in Table 3 and across the overlength classes in Table 4.

We also had the annotators annotate some overlapping images over the three sequences. In Seq1 and Seq2 a total of 10 and 5 images were annotated, respectively, by all three annotators. Whereas in Seq3, 5 images were annotated by both annotators 1 and 2. An analysis of the inter-rater agreement using Cohen’s Kappa coefficient [43] confirms that there is little agreement, as seen in Table 5. Cohen’s Kappa is a statistic that can measure the reliability of two persons annotating the same instances while taking into account that the agreement could be by chance. We define an annotation to be an agreement when two polygon annotations have an Intersection-over-Union (IoU) greater than 0.5. Table 5 shows that for each sequence pair, the agreement scores 0, which can be interpreted as no agreement. Additionally, in the right portion of the table we show for a given annotator the number of annotated instances and the number of agreed annotations per counterpart annotator.

Based upon the above observations we perform an additional experiment to highlight the potential pitfalls of using inconsistent annotations by training two different models. Firstly, we focus on how well the model performed in terms of precision and recall, as well as on what effect this has when evaluating with a test set if annotations are not consistent in training. It can be challenging to optimise a model when annotations are not consistent. However, it is also difficult to determine if alterations to a model improve or worsen if the basis of false positives and true positives are incorrect during testing. Therefore, models were trained on two datasets of different consistency, namely a Faster R-CNN [44] with an Inceptionv2 [45] backbone using transfer learning from COCO using the TensorFlow Object Detection API [46]. This is the same training strategy used for baseline overlength models in [2].

In Table 6 we show Average Precision (AP) and Average Recall (AR) results based on COCO standards [5] on a test set with inconsistent annotations. For each metric we show two values, the upper being a model trained on inconsistent annotations from all three annotators and the lower trained on consistent annotations from Annotator 3. In both cases the annotations are split 70% for training, 15% for validation, and 15% for testing. Additionally, the splits from Annotator 3 were the same across both datasets to ensure comparable results. Table 6 shows when looking at all classes that the model trained on consistent data performs in general a number of percentage points (p.p.) higher but scores lower on AR when more predictions are allowed. There is a clear difference between the two models when evaluating inner stalk predictions. Here, the model trained on consistent data scores between 20 to 30 p.p. higher.

Clearer results can be seen when evaluating on the consistent test set in Table 7. Increases in AP can be seen for the consistent-trained model, with AP@0.75 rising by almost 15 p.p. For individual classes significant increases are seen for all classes except for the outer stalks in terms of AP.

Table 6 and Table 7 show the importance of having consistent data not only when training models but also when evaluating them. In both tables it can be seen that in general the model trained on consistent annotations have a higher AP compared to the inconsistent counterpart. Additionally, for the inconsistent model in Table 7, the AP metrics are increased significantly in comparison to Table 6. Therefore, if the model was evaluated on inconsistent annotations a conclusion could be made that the model performs poorly.

## 4. Semi-Supervised Learning

Due to the challenges and inconsistencies between annotators we perform investigations into the potential of using SSL to complement manual annotation for our dataset. We adopt the Unbiased Teacher methodology [31] due to their recent improvements with SSL for object detection. SSL has not been as extensively used in object detection tasks in comparison to classification, as there is often a significant bias towards background in comparison to foreground. Therefore, the usage of pseudo labelling between a teacher and student network can be prone to learning a bias towards easier objects. However, with the Unbiased Teacher [31], the authors identify that in two-stage recognition networks, such as Faster R-CNN, overfitting occurs in the classification heads for both the Region Proposal Network and final multi-class classification. The approach proposes to train a student and teacher mutually, where the student learns from the teacher via highly augmented images and the teacher learns slowly from the student with an Exponential Moving Average (EMA). In addition to EMA, the framework adopts focal loss to concentrate on more challenging examples in order to lower the bias towards easier examples. The framework has a number of parameters that must be tuned in order to allow the two networks to improve together. Firstly, a confidence threshold that defines which predictions from the teacher are passed as annotated examples to the student. Second, the number of unsupervised images per iteration to create pseudo labels form a variable controlling how much weight the unsupervised examples have when calculating loss. Finally, a number of burn-in iterations must be set, where the teacher network is trained in order to provide a solid baseline before performing SSL.

We evaluate the usage of SSL by training teacher-student networks on two different annotated datasets together with a large number of unannotated images for kernel fragmentation. This includes the 151617 dataset presented earlier and used in a number of previous works [1,2,15] and a subset only including annotations from 2016. The 151617 training set includes 1393 images containing 6907 instances and the 2016 subset has 115 images with 675 instances. Our unsupervised portion of the SSL dataset contains 7888 images from a harvest captured in 2019. Finally, we evaluate our SSL-trained models with both object detection metrics and correlation analysis against physically sieved CSPS samples first presented in [2]. For a stronger evaluation we use a new test set compared to previous WPCS works, adopting the sanity checked annotations from 2018 presented in Table 1 and Figure 7. This way we allow for less precise annotations during the training process but test networks against annotations of higher quality.

Our teacher-student networks follow the investigations done in [31] which are a Faster R-CNN [44] with an ResNet50 [47] Feature Pyramid Network [48] backbone. Networks are trained on an NVIDIA Titan XP GPU using the Detectron2 framework [49]. The pseudo-code for training our networks using SSL can be seen in Algorithm 1. The networks are trained for at total of 50,000 iterations with a learning rate of 0.01 using Stochastic Gradient Descent. An initial burn-in of 10,000 iterations trains on the set of supervised images and then the network is duplicated into a teacher and student variant. Then, at each training iteration, by using a number of unsupervised images, the teacher first generates pseudo-labels on images with weak augmentation. These labels are then used to train the student network on the same set of images but with strong augmentation. Finally, the teacher network is refined with the weights from the student using an EMA of 0.9996. A lower EMA would allow the student to contribute more during updates of the teacher network and may cause worse performance due to too noisy labels [31]. Finally, we also train baseline Faster R-CNN models in a fully supervised manner to compare our SSL models against.
**Algorithm 1:** Teacher-student training overview1:Train model on supervised set in burn-in step for 10,000 iterations2:After burn-in duplicate model into teacher and student3:**for** each training iteration on a set of unsupervised images **do**4:       Teacher generates pseudo-labels on images with weak augmentation5:       Student uses pseudo-labels to update network with strong augmentation6:       Teacher network refined using EMA from update student network7:**end for**

In Table 8, the results can be seen for a number of different teacher-student variants. Additionally, the baseline model can be seen in the first row where the parameters for SSL are not applicable. The remaining rows show how SSL training runs with all combinations of the three key SSL parameters. The confidence threshold for pseudo labels is set between 0.1 to 0.7 with 0.2 increments. The number of unsupervised images per iteration and how much weight to place on the unsupervised loss is set at either 1 or 4. For each SSL-trained model we evaluate the AP and Pearson’s Correlation Coefficient (PCC) with the network iteration with the lowest validation loss. Also shown in the table is that two SSL training runs diverged early and therefore results are not shown. In regards to AP metrics we see that SSL models trained with a bounding-box confidence threshold of either 0.3 or 0.5 improve results in comparison to the baseline model. The best performing model for AP and AP@0.5 are seen with a confidence threshold of 0.5, using 4 unsupervised images and an unsupervised weight of 4. Concretely, the AP is improved by 3.55 p.p. and AP@0.5 by 6.2 p.p. At the more stringent AP@0.75 the network trained with the same parameters apart from a confidence threshold of 0.3, has a slight improvement with 4.51 p.p. The PCC analysis can be seen in the three right-most columns in Table 8 and we see that the best performing models for AP does not translate to improvements in PCC. However, the PCC is improved for CW43 by 0.04 and when combining the two harvest weeks by 0.07.

In Table 8, we applied SSL in combination to the 151617 dataset, which required a relatively large amount of effort in obtaining the initial 6907 annotated object instances. Therefore, in Table 9 we investigate whether much less effort can be used, and therefore only use the annotations from 2016 containing 675 instances. The unsupervised portion is extended to also include the images from 2015 and 2017 from the 151617 dataset. This means that 1.4% of the dataset in Table 9 is annotated, compared to 15.1% in Table 8. The baseline model shows a considerable drop in AP and PCC in comparison to previous results. For example, AP decreases by 12.23 p.p. and 15.78 p.p. to 4.97 in comparison to the baseline and best performing model using 151617 as supervised labels. However, the teacher-student training with additional unsupervised data improves the baseline by a large margin. The SSL model trained with 0.7 confidence threshold, 4 unsupervised images, and an unsupervised weight of 4, which increases AP by 12.69 p.p., AP@0.5 by 26.52 p.p., and AP@0.75 by 10.19 p.p..The same model increases the PCC for both harvest weeks from 0.56 to 0.64. However, this improvement appears to be present largely for the first week as better PCC can be seen for another teacher-student training at 0.1 bounding-box threshold. Overall, the AP and PCC results are not improved in comparison to those in Table 8, however, significant effort in the annotation could have been saved using this approach.

## 5. Discussion

Despite implementing annotation guidelines and using subject-matter experts as annotators, we founds variation and inconsistencies. This shows both the difficult task of annotating our images and of manual annotation in general. The annotations could likely be improved with increased processes such as multiple annotation iterations per image/harvest and gold standard sets. However, these could be costly to implement and also be time-consuming. We investigated a single alternative to manual annotation through a teacher-student training framework. Others could be of interest, such as an annotation tool aiding through automatic annotation.

Despite annotation inconsistency, we still see a strong correlation in our models and in previous work. Therefore, we suggest that the dataset is still suitable for training, but care should be taken when evaluating models with annotation-based metrics such as AP. Instead, this should be done in conjunction with physically-sieved estimates such as CSPS.

**Figure 10:**
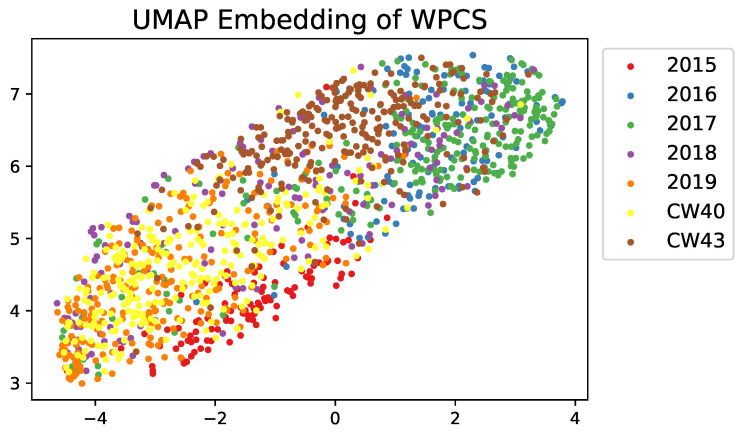
UMAP embeddings of various RGB images captured during different harvests.

In our datasets, especially for kernel fragments, bias has been attempted to be counteracted by including images from multiple different harvest seasons and machine settings. There is likely considerable variation in WPCS and the resulting images. If a system were to be evaluated across thousands of farms all over the world, care should be taken for additional datasets to take this into account. For examples, in Figure 10 we show the UMAP [50] embeddings of a random sample of up to 250 images from our images over the multiple harvests. We see that the RGB embeddings do cluster and this information could be utilised.

## 6. Conclusions

The majority of deep learning methods are reliant on annotation. This can be difficult and expensive for more specific applications, such as within agriculture. The annotation process is often not covered in such datasets, making it difficult to reproduce or evaluate the research fully. Therefore, the aim of this work was to describe a concrete case and thereby illustrate the actual challenges and how we have addressed them.

In this work we have presented for WPCS our annotation process, statistics, and an analysis of our datasets, which is not often done in specific use-cases within agriculture. Manual annotation is often a challenging and time-consuming task, which has also been the case in our dataset, as seen by variations in statistics for the annotations between annotators and between harvest seasons.

We evaluate the usage of SSL, with a teacher-student approach as an extension to manual annotations. Our SSL-trained object detectors showed promise by increasing AP, but showed no significant alteration when evaluating CSPS against physical samples. However, we did see significant improvements when using the approach on a much smaller annotated set from a single harvest season.

Given that we have covered and gained knowledge on the challenges within WPCS annotation, further research on how to improve the overall annotation quality should be conducted. Performing an incremental implementation of some additional processes seen in larger benchmarks could uncover which would be beneficial while hopefully keeping costs reasonable. Tools allowing for assisted annotation can alleviate some of the feedback we received from annotators, specifically that it can be difficult to see differences between objects of interest and background, and that the process is too repetitive, leading to issues with concentration. Furthermore, adding extra supervision and gold standards could quickly localise potential errors. These steps will allow for stronger data, required for better training of models and evaluation against annotated test sets.

We hypothesise that a combination of increased processes and further alternative tools can significantly decrease the annotation cost, as larger datasets would be required to cover additional variations in farms. We believe that exploring challenges in smaller datasets is a crucial step in all domains. Being aware or addressing them to improve the overall quality is crucial for success, whether it be training successful models or having the ability to evaluate them with annotation-based metrics.

## Figures and Tables

**Table 1:** Annotation statistics for the images captured over four different harvest seasons.

PG	TLOC	Images	Anno Insts	Insts per Img
2015				
1 (2)	9	90	1333	14.8
2 (1)	9	21	189	9.0
3 (1)	9	37	402	10.31
4 (1)	9	39	300	8.11
Total		187	2224	11.89
2016				
1 (14)	4	131	762	5.82
2 (2)	4	18	110	6.11
3 (2)	4	19	82	4.32
4 (1)	4	11	58	5.27
Total		205	1118	5.45
2017				
1 (2)	4	152	967	6.36
2 (2)	4	127	458	3.61
3 (2)	4	359	901	2.51
3.5 (2)	4	126	442	3.51
1 (2)	12	290	1200	4.14
2 (2)	12	289	1909	6.61
3 (2)	12	111	927	8.35
3.5 (2)	12	171	435	2.54
Total		1972	8270	4.19
2018				
1 (1)	6	20	616	28.00
2 (1)	6	20	567	25.77
3 (1)	6	20	507	25.35
4 (1)	6	20	472	23.60
1 (1)	11.5	20	448	22.40
2 (1)	11.5	20	361	18.05
3 (1)	11.5	20	238	11.90
4 (1)	11.5	20	264	10.56
Total		169	3473	20.55

**Table 2:** Annotation statistics for the overlength dataset from [2].

TLOC	Images	Instances	A Leaves	NA Leaves	Inner Stalk	Outer Stalk	Avg. Size	Avg. Major Axis Length	Avg. Minor Axis Length
4	163	1233	520	419	75	209	14,518.9	216.6	94.3
6	199	904	182	559	35	122	26,315	294.3	122.7
11.5	113	263	51	172	1	38	61,328.2	485.5	179.9

**Table 3:** Annotation statistics for overlengths for three different annotators. Each numbered sequence contains images harvested with the same machine settings.

	Images	Instances	Avg. Insts per Image	Avg. Size	Avg. Major Axis Length	Avg. Minor Axis Length
Annotator 1						
Seq1	37	73	1.97	33,056.85	322.33	140.05
Seq2	32	57	1.78	36,415.78	360.33	124.02
Seq3	31	102	3.29	25,180.66	292.98	124.02
Annotator 2						
Seq1	37	124	3.35	25,423.53	294.71	126.33
Seq2	32	180	5.62	20,969.54	262.65	111.25
Annotator 3						
Seq1	37	271	7.32	17,105.99	234.34	102.44
Seq2	32	256	8.0	18,098.88	232.60	111.25
Seq3a	31	227	7.32	18,025.16	234.55	111.41
Seq3b	31	222	7.16	18,427.43	242.06	110.28

**Table 4:** Class instances annotated by the three annotators for stover overlengths.

Annotator	A Leaves	NA Leaves	I Stalks	O Stalks
Annotator 1	122	46	7	7
Annotator 2	82	98	10	24
Annotator 3	418	330	65	157

**Table 5:** Cohen Kappa Score between each annotator pair with a single annotator as reference annotator (left-most column). Additionally, the total number of instances per reference annotator with counts of overlap where IoU is greater than 0.5 for each sequence.

Cohen Kappa			Count IoU > 0.5		
A1	A2	A3	Inst Cnt A1	A2	A3
Seq1	0	0	25	1	0
Seq2	0	0	6	0	0
Seq3	0	na	15	na	15
A2	A1	A3	Inst Cnt A2	A1	A3
Seq1	0	0	62	7	4
Seq2	0	0	23	3	1
Seq3	na	na	na	na	na
A3	A1	A2	Inst Cnt A3	A1	A2
Seq1	0	0	78	9	6
Seq2	0	0	37	4	3
Seq3	0	na	39	3	na

**Table 6:** Results on a test set with inconsistent annotations from the three annotators. For each metric, results from two models are shown trained on different sets of data, the upper being trained on the inconsistent dataset and the lower being trained on the consistent dataset.

Class	AP	AP@0.5	AP@0.75	AR@1	AR@10	AR@100
All (207)	23.7	42.2	25.8	23.0	42.5	47.8
	28.1	48.1	34.8	26.9	42.1	45.5
A Leaves (107)	29.1	47.3	33.6	17.4	51.6	57.8
	29.2	41.8	39.6	17.7	55.7	61.3
NA Leaves (59)	17.9	34.2	17.4	19.3	35.3	44.4
	20.0	34.2	21.2	22.8	30.6	35.6
I Stalks (11)	31.7	54.7	31.3	27.3	50.9	50.9
	51.7	76.3	59.2	34.6	51.8	55.4
O Stalks (30)	15.9	32.7	20.8	28.0	32.3	38.0
	10.0	30.6	19.0	32.3	23.3	29.7

**Table 7:** Results on a test set with consistent annotations from the one annotator. For each metric, results from two models are shown trained on different sets of data, the upper being trained on the inconsistent dataset and the lower being trained on the consistent dataset.

Class	AP	AP@0.5	AP@0.75	AR@1	AR@10	AR@100
All (141)	32.0	54.2	35.6	23.8	45.1	49.1
	39.7	63.0	50.4	29.6	46.0	46.9
A Leaves (64)	44.6	70.7	54.7	20.2	55.8	61.1
	49.1	70.8	68.5	22.6	60.8	63.9
NA Leaves (43)	23.5	45.0	19.6	17.2	35.6	43.5
	27.8	48.7	25.5	23.7	29.3	34.4
I Stalks (10)	37.4	64.5	36.9	30.0	56.0	56.0
	60.5	97.5	69.0	38.0	68.0	61.0
O Stalks (24)	22.5	36.6	31.3	27.9	32.9	35.8
	21.1	35.1	38.5	34.1	25.8	28.3

**Table 8:** Results for SSL-trained models for models with various hyper-parameters on the 151617 annotated dataset together with unannotated images from a harvest from 2019. Additional results are also shown for baseline fully supervised models in the first rows.

Train Set	Unsup. Set	Bbox Thresh	Unsup Images	Unsup Weight	AP	AP@0.5	AP@0.75	PCC CW40	PCC CW43	PCC CW40+43
151617 [2]	NA	NA	NA	NA	NA	NA	NA	**0.95**	**0.79**	**0.81**
151617	NA	NA	NA	NA	17.20	32.15	15.96	0.94	0.75	0.68
151617	2019	0.1	1	0.5	15.57	27.73	16.16	0.88	0.73	0.72
151617	2019	0.1	1	4	-	-	-	-	-	-
151617	2019	0.1	4	0.5	17.49	31.36	17.40	0.86	0.76	0.71
151617	2019	0.1	4	4	-	-	-	-	-	-
151617	2019	0.3	1	0.5	17.85	31.92	17.78	0.93	0.72	0.75
151617	2019	0.3	1	4	19.93	36.02	19.64	0.81	0.74	0.67
151617	2019	0.3	4	0.5	17.95	32.32	17.66	0.92	0.71	0.72
151617	2019	0.3	4	4	19.73	35.15	**20.47**	0.85	0.69	0.65
151617	2019	0.5	1	0.5	19.79	35.86	20.32	0.90	**0.79**	0.70
151617	2019	0.5	1	4	17.78	34.85	15.45	0.83	0.73	0.62
151617	2019	0.5	4	0.5	19.66	36.45	18.54	0.88	0.72	0.65
151617	2019	0.5	4	4	**20.75**	**38.35**	19.99	0.88	0.72	0.63
151617	2019	0.7	1	0.5	15.56	27.82	15.36	0.88	0.63	0.63
151617	2019	0.7	1	4	15.36	28.13	15.13	0.77	0.58	0.59
151617	2019	0.7	4	0.5	15.47	28.48	14.84	0.86	0.60	0.58
151617	2019	0.7	4	4	13.58	24.37	13.22	0.77	0.55	0.56

**Table 9:** Results for SSL-trained models for models with various hyper-parameters on the 2016 annotated dataset together with unannotated images from harvests from 2015, 2017, and 2019. Additional results are also shown for baseline fully supervised models in the first rows.

Train Set	Unsup. Set	Bbox Thresh	Unsup Images	Unsup Weight	AP	AP@0.5	AP@0.75	PCC CW40	PCC CW43	PCC CW40+43
2016	NA	NA	NA	NA	4.97	7.39	6.05	0.70	0.54	0.56
2016	1517+2019	0.1	1	0.5	11.95	24.02	9.65	0.72	0.65	0.63
2016	1517+2019	0.1	1	4	-	-	-	-	-	-
2016	1517+2019	0.1	4	0.5	14.24	28.51	11.51	0.70	**0.76**	0.59
2016	1517+2019	0.1	4	4	12.00	22.43	11.32	0.74	0.66	0.65
2016	1517+2019	0.3	1	0.5	13.67	27.15	10.89	0.70	0.57	0.52
2016	1517+2019	0.3	1	4	-	-	-	-	-	-
2016	1517+2019	0.3	4	0.5	13.28	27.90	9.35	0.85	0.55	0.62
2016	1517+2019	0.3	4	4	13.53	24.15	13.31	0.84	0.66	0.70
2016	1517+2019	0.5	1	0.5	15.05	29.60	12.30	0.73	0.64	0.56
2016	1517+2019	0.5	1	4	-	-	-	-	-	-
2016	1517+2019	0.5	4	0.5	16.98	33.60	13.98	0.83	0.70	**0.71**
2016	1517+2019	0.5	4	4	16.62	32.75	14.16	0.79	0.65	0.58
2016	1517+2019	0.7	1	0.5	12.34	21.14	13.52	0.82	0.55	0.64
2016	1517+2019	0.7	1	4	13.92	27.88	11.91	0.85	0.61	0.58
2016	1517+2019	0.7	4	0.5	9.67	16.23	10.59	0.74	0.59	0.64
2016	1517+2019	0.7	4	4	**17.66**	**33.91**	**16.24**	**0.90**	0.61	0.64

## Data Availability

Not applicable.

## Abbreviations

The following abbreviations are used in this manuscript:

WPCS	Whole Plant Corn Silage
CNN	Convolutional Neural Network
SSL	Semi-Supervised Learning
PG	Processor Gap
TLOC	Theoretical Length of Cut
CSPS	Corn Silage Processing Score
IoU	Intersection-over-Union
AP	Average Precision
AR	Average Recall
p.p.	Percentage Points
EMA	Exponential Moving Average
PCC	Pearson’s Correlation Coefficient
